# Use of Electronic Health Record Access and Audit Logs to Identify Physician Actions Following Noninterruptive Alert Opening: Descriptive Study

**DOI:** 10.2196/12650

**Published:** 2019-02-07

**Authors:** Azraa Amroze, Terry S Field, Hassan Fouayzi, Devi Sundaresan, Laura Burns, Lawrence Garber, Rajani S Sadasivam, Kathleen M Mazor, Jerry H Gurwitz, Sarah L Cutrona

**Affiliations:** 1 Meyers Primary Care Institute Worcester, MA United States; 2 University of Massachusetts Medical School Worcester, MA United States; 3 Reliant Medical Group Worcester, MA United States; 4 University of Massachusetts Memorial Health Care Worcester, MA United States; 5 Edith Nourse Rogers Memorial Veterans Hospital Veterans Health Administration Bedford, MA United States

**Keywords:** electronic health records, health services research, health information technology, health care communication

## Abstract

**Background:**

Electronic health record (EHR) access and audit logs record behaviors of providers as they navigate the EHR. These data can be used to better understand provider responses to EHR–based clinical decision support (CDS), shedding light on whether and why CDS is effective.

**Objective:**

This study aimed to determine the feasibility of using EHR access and audit logs to track primary care physicians’ (PCPs’) opening of and response to noninterruptive alerts delivered to EHR InBaskets.

**Methods:**

We conducted a descriptive study to assess the use of EHR log data to track provider behavior. We analyzed data recorded following opening of 799 noninterruptive alerts sent to 75 PCPs’ InBaskets through a prior randomized controlled trial. Three types of alerts highlighted new medication concerns for older patients’ posthospital discharge: information only (n=593), medication recommendations (n=37), and test recommendations (n=169). We sought log data to identify the person opening the alert and the timing and type of PCPs’ follow-up EHR actions (immediate vs by the end of the following day). We performed multivariate analyses examining associations between alert type, patient characteristics, provider characteristics, and contextual factors and likelihood of immediate or subsequent PCP action (general, medication-specific, or laboratory-specific actions). We describe challenges and strategies for log data use.

**Results:**

We successfully identified the required data in EHR access and audit logs. More than three-quarters of alerts (78.5%, 627/799) were opened by the PCP to whom they were directed, allowing us to assess immediate PCP action; of these, 208 alerts were followed by immediate action. Expanding on our analyses to include alerts opened by staff or covering physicians, we found that an additional 330 of the 799 alerts demonstrated PCP action by the end of the following day. The remaining 261 alerts showed no PCP action. Compared to information-only alerts, the odds ratio (OR) of immediate action was 4.03 (95% CI 1.67-9.72) for medication-recommendation and 2.14 (95% CI 1.38-3.32) for test-recommendation alerts. Compared to information-only alerts, ORs of medication-specific action by end of the following day were significantly greater for medication recommendations (5.59; 95% CI 2.42-12.94) and test recommendations (1.71; 95% CI 1.09-2.68). We found a similar pattern for OR of laboratory-specific action. We encountered 2 main challenges: (1) Capturing a historical snapshot of EHR status (number of InBasket messages at time of alert delivery) required incorporation of data generated many months prior with longitudinal follow-up. (2) Accurately interpreting data elements required iterative work by a physician/data manager team taking action within the EHR and then examining audit logs to identify corresponding documentation.

**Conclusions:**

EHR log data could inform future efforts and provide valuable information during development and refinement of CDS interventions. To address challenges, use of these data should be planned before implementing an EHR–based study.

## Introduction

Audit and access logs in the electronic health record (EHR) have primarily been used for security purposes [1-3], but recent studies [4-7] indicate that a broad range of additional research and clinical questions may be answered using this relatively untapped data source. Access logs contain time-stamped recordings of who accessed the EHR and what part was accessed [5]. Audit logs record chronological activity in the EHR, tracking actions such as what data were created, reviewed, or changed by the user [3]. These log data, though potentially complex to retrieve and interpret, can be used by researchers and care teams to identify targets for clinical interventions as well as care quality and safety-improvement efforts [4,8].

Data from access and audit logs can be used to better understand how various forms of clinical decision support (CDS) impact physician behavior. This is especially relevant for noninterruptive EHR alerts, where assessments of the effectiveness of these alerts have found mixed results [9-12]. In contrast to alerts that “pop-up” and interrupt workflow when a specific EHR action is taken, noninterruptive alerts deliver information to an EHR InBasket, requiring the receiver to open and review them. This information may include warnings about out-of-range test results [9,13,14], abnormal findings on diagnostic imaging and screening [10,15-17], important changes in patient health [18], and safety concerns during transition of care [19]. However, these alerts must compete for the attention of physicians who are often overwhelmed by the number of electronic notifications they receive, read, and respond to while managing direct patient care [4,20-23].

Access logs can provide valuable insight into the precise time of alert opening, even if the alert is opened many days postdelivery; logs can also help pinpoint actions following opening that might otherwise be difficult to link back to a particular CDS intervention. Although causality of EHR actions can be difficult to prove, there is value to demonstrating close temporal proximity of an EHR action after alert opening. Such knowledge can shed light on the mechanisms by which an EHR–based intervention is (or is not) effective.

The effectiveness of a noninterruptive alerting system was tested by our team in a previous randomized trial (trial registration: ClinicalTrials.gov NCT00611091) [19]. Locally developed alerts regarding older adults’ posthospital discharge were sent to their primary care physicians (PCPs) through the EHR [24,25]. These alerts highlighted what our team believed to be actionable medication safety concerns. The trial assessed whether these alerts increased outpatient visits and reduced rehospitalization [19]. The alerts did not improve either of these measures. To better understand this outcome, we conducted this descriptive study using audit and access log data to track actions taken by physicians in the EHR following alert delivery. In this paper, we present findings from this analysis of physician action. We also describe the availability and level of detail of information in these logs and review challenges and strategies for success.

## Methods

### Design

This study was based on data from the intervention arm of a prior randomized controlled trial, as mentioned above [19]. In that trial, an EHR–based intervention was implemented using locally developed noninterruptive alerts to highlight postdischarge medication safety concerns for older patients. The inpatient facility to which the health care system admitted its patients used a different EHR than that used by the health care system. An interface engine was linked to the hospital’s admission, discharge, and transfer registration system. The hospital transmitted information including discharge dates for the health care system’s patients; these data were automatically incorporated into the health care system’s EHR. Informed by health plan data reflecting new prescriptions filled, alerts were then generated for patients. These alerts were designed to convey actionable time-sensitive concerns pertaining to high-risk drugs (known to result in more adverse drug events) and new drugs. Over the 4 months prior to implementation, two physicians from the health care system met weekly to review every alert generated, suggesting modifications to ensure that alerts would be perceived as necessary, useful, and brief. Alerts that they considered inactionable were eliminated. At the time of hospital discharge, patients were randomized to the intervention (EHR alerts sent to the patient’s PCP if medication concerns were identified) or usual follow-up care. The primary goal of the intervention was to decrease rehospitalization rates.

### Setting

This study was conducted at a large Massachusetts multispecialty medical group with 217 physicians at 15 sites throughout Central Massachusetts. All sites used an EHR from Epic Systems Corporation.

### Study Sample

When the original trial was conducted in 2011, approximately 140,000 adults received primary care through this medical group, of which approximately 24,000 were aged ≥65 years and were members of a local health plan. Patients who were members of this local health plan with primary care providers within this medical group and were discharged to home from the primary hospital used by the medical group from August 26, 2010, to August 25, 2011, were included in the trial. A total of 1282 patients were randomized to the intervention arm. In our analysis, we included only the 799 patient discharges for which PCP medication alerts were triggered (corresponding to 713 patients). These alerts were sent to 75 PCPs.

The Institutional Review Boards at Reliant Medical Group and the University of Massachusetts Medical School approved this study, and waiver of consent was obtained.

### Variables and Data Sources

Data available from the original trial included details about the timing and content of alerts, the scrambled identifier of the physician to whom the alerts were sent, and additional PCP characteristics including age (<50 vs ≥ 50 years), gender, and specialty (internal medicine, family medicine, PCP without a Doctor of Medicine degree such as a nurse practitioner, and subspecialist acting as PCP). To approximate full- versus part-time status of the physicians, we categorized the total number of patient encounters for each physician during the year prior to alert delivery in quartiles (0-2326, 2327-2783, 2784-3173, and >3173) across the 75 PCPs to whom alerts were sent. We collected the following information about the relevant patients: age (65-74, 75-84, and ≥85 years), gender, Charlson comorbidity index score (categorized as 0, 1, 2, and ≥3), dates and length (≤2 days, 3 days, and ≥4 days) of the related hospitalization, number of office visits in the past year (≤6, 7-11, 12-18, and >18), patient outcomes, and scrambled patient identifiers. Our research team grouped all the alerts sent during the intervention into three categories: information about new and high-risk medications at the time of hospital discharge (“information only”), recommendation to cancel or modify doses of medications (“medication recommendation”), and recommendation to order tests—laboratory tests or, in a few cases, eye exams—to monitor the impact of high-risk medications or titrate their doses (“test recommendation”).

For this descriptive study, we sought a range of additional data elements from the EHR access and audit logs (described below) to provide information on the opening of alerts and track specific physician actions within the EHR. We captured the timing of alert opening relative to alert delivery (≤24 hours, 24-48 hours, and >48 hours) within office hours (8 AM-5 PM Monday through Friday) as compared to outside office hours. We also identified the user opening the alert (the intended PCP vs a staff member or provider other than the intended PCP).

We gathered data on the following variables pertaining to the PCP’s InBasket load categorized by quartile: total number of notifications in the InBasket at the time of alert delivery (≤42, 43-69, 70-157, and >157), number of unopened notifications in the InBasket at the time of alert delivery (0, 1-4, 5-9, and >9), and number of notifications delivered to the InBasket in the 7 days prior to alert delivery (≤344, 345-453, 454-546, and >546). Finally, we compared alerts delivered on Saturdays to those delivered on all other days, because those arriving on Saturday were the only alerts for which the 24 hours postdelivery did not include any weekday time.

Our goal was to identify factors associated with the alerts that impacted physician review of patient information (eg, review of EHR information related to prescribed medications, laboratory test results, and laboratory orders) or physician orders for medications or laboratory tests.

### Identifying Primary Care Physicians’ Actions in the Electronic Health Record Through Use of Access and Audit Logs

We analyzed EHR log data corresponding to the 2-day period following opening of 799 alerts (593 information only, 37 medication recommendations, and 169 test recommendations) sent to 75 PCPs’ InBaskets. These three types of alerts highlighted new medication concerns for older patients posthospital discharge; all were intended to prompt review of the recently hospitalized patient’s chart within the EHR by the PCP. Expected actions for test recommendations included viewing test results or ordering tests. Expected actions for medication recommendations included viewing medication lists and discontinuing or ordering medications. For information-only alerts, appropriate action was left to the judgment of the PCP, but expected actions were similar to those for medication recommendations and included performance of medication reconciliation, which would entail viewing medication lists and possibly discontinuing or ordering medications. In all cases, reviews of other parts of the patient chart could be expected in support of information gathering and decision making.

In order to retrieve data on the 2-day period following alert opening, the data manager needed to first identify the location and format of data generated by the locally developed alert system. This work was accomplished through careful coordination between a physician with EHR access and a data manager. Simulated patients were created, and alerts were triggered for these simulated patients. Following this, a collaborating physician from the medical group opened the alerts and then opened and viewed various sections within the EHR corresponding to the simulated patient’s chart. The data manager used the collaborating physician and simulated patient identifiers together with the dates and times of triggering activities to locate these actions within the EHR’s logs and tables. This allowed her to identify the code generated by the locally developed alert system, indicating that an alert had been sent. From that base, the unique identifiers generated for each delivered alert could be extracted. Using the audit log table, we then obtained time stamps for each alert opened (hour, minute, and second) and an identifier for the person opening the alert in order to track actions. Alerts could be opened by the PCP to whom they were addressed or other members of the care team.

### Immediate Actions

We identified the first time the alert was opened by the addressed PCP. The InBasket view of the EHR version used during the study period only displayed the alert message and several clickable buttons that served as direct links to summaries of sections of the corresponding patient’s chart; thus, navigation to other sections of the EHR was required to gain any additional information. After opening the alert, some PCPs navigated to sections of the EHR for the patient triggering the alert, whereas some moved on to a different alert or the electronic record for a different patient. Using time stamps, we captured the time spent by the PCP on the alert itself, and we captured the total time spent on the relevant patient. Total time included the duration during which the alert was displayed in the EHR plus the time the PCP spent navigating sections of the EHR corresponding to the relevant patient.

Using the EHR access log table, we identified each action taken by the provider during the 5-minute period immediately following alert opening. Data elements from each action provided information on the exact time (hour, minute, seconds) of the action as well as codes indicating the specific section of the EHR opened and the patient to whom it related. Codes indicating the component of the EHR accessed by the PCP were complex, requiring additional physician-data manager collaboration to categorize. Actions that we considered as viewing relevant patient information included opening a section of the electronic medical record (medications, laboratory, orders, results, encounters, demographic information, other clinical information, nonclinical information, and information entry) or choosing one of several options on the alert that served as direct links to summaries of components of that patient’s record. Actions that we considered as not viewing relevant patient information included opening a notification related to a different patient, opening a section of a different patient’s medical record, or doing nothing further in the EHR for 5 minutes.

### Subsequent Relevant Actions

Considering that PCPs might be opening alerts briefly in between patient visits and might return to the patient record later to address issues raised in the alert, we sought to broaden the window for tracking PCP’s actions. For our analysis of subsequent relevant actions taken by the PCP, the time window studied included the day of alert opening and the following day. Extracted data included a timestamp as well as codes indicating the EHR component accessed (categorized in the same manner as with immediate action analyses described above). In addition to the information extracted from the access log table, we captured evidence of orders placed for medications (new medication, change, or discontinuation) and laboratory tests. We focused only on actions taken in the EHR for the patient of interest (eg, the patient for whom the alert was triggered).

We categorized PCP actions under the broad heading of general action (includes viewing any patient data, creating documentation, and placing any orders). Within this broad group, we defined subcategories: medication-specific action (viewing the patient’s medication list or ordering a medication) and laboratory-specific action (viewing or ordering laboratory tests).

### Contextual Factors

We also examined contextual factors potentially relevant to alert opening and subsequent EHR action. These included the number of messages and unopened messages in the physician’s InBasket as well as the flow of messages during the prior week. Capturing a snapshot of the EHR status at a historical point in time (eg, number of InBasket messages at the time of alert delivery) presented challenges. To recreate the InBasket at the moment of alert delivery, we began by assembling all the messages delivered to a specific physician’s InBasket during the 1-year period prior to alert delivery. We then eliminated any messages for which the record indicated that the message was “completed” (eg, deleted from the InBasket) prior to the date of alert delivery. This provided a count of the number of messages remaining in the InBasket. To calculate the number of messages that remained unopened at the time of alert delivery, we identified those for which there was no log entry indicating opening on or prior to the alert delivery date. We also calculated the total number of notifications that arrived in the InBasket during the week prior to alert delivery.

### Data Analysis

#### Immediate Actions

For assessment of the immediate response during the 5 minutes after alert opening, we focused only on those cases in which the physician to whom the alert was sent was the first person to open the alert. We categorized the immediate next action by the physician as one related or not related to the relevant patient. We used bivariate analyses to assess the relationship between performing an immediate relevant action and alert type, timing of alert opening relative to alert delivery, provider and patient characteristics, and contextual factors. We performed multivariate analyses to obtain odds ratios (OR) in the presence of more than one variable. Since some PCPs received multiple alerts over the 1-year period, we used generalized estimating equations with a logit link and a binomial distribution to account for clustering of measures within PCPs. To estimate the total time attributable to the alert in the 5 minutes following opening, we calculated the length of time spent by the PCP with the alert open (ie, displayed on the EHR computer screen) and combined this with the total time (immediately following alert opening) spent viewing sections of the EHR for that patient.

#### Subsequent Relevant Actions

When assessing factors related to relevant subsequent actions, we included all first openings of each alert irrespective of whether the addressed physician or a covering physician or staff member opened the alert, but tracked behavior of the addressed physician. Similar to the process for the immediate actions, we performed bivariate and multivariate analyses using generalized estimating equation models to assess the relationship between performing subsequent relevant actions and the covariates mentioned above. We also assessed the effect of alert opening by the addressed physician on the likelihood of subsequent actions.

## Results

### Overview

**Figure 1:**
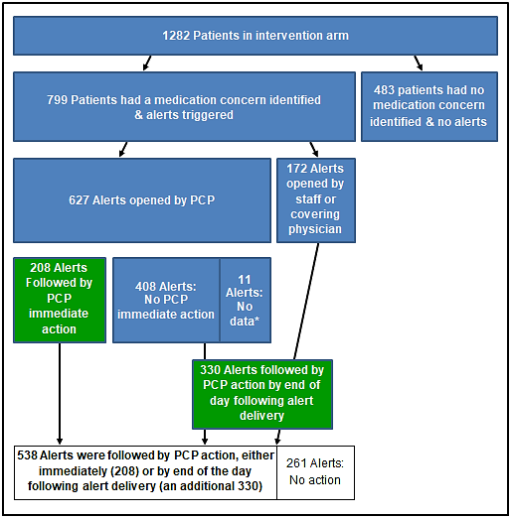
Overview of alert opening. Asterisk indicates that 11 alerts had no data available on immediate action. PCP: primary care physician.

In the overall group of 799 alerts opened, 627 (78.5%) were opened by the addressed PCP. Of this subgroup, we were able to track actions immediately following PCP alert opening for 616 alerts (11 alerts had no available data). The analysis was then expanded to include the remaining 172 (21.5%) alerts opened by staff or a provider other than the PCP to track actions over the day of alert opening and the following day. Alerts that did not show evidence of action within this timeframe were classified as alerts not followed by timely PCP action (Figure 1).

### Immediate Actions

Among the 616 tracked alerts, 208 (33.8%) were immediately followed by viewing of the relevant patient’s EHR (Table 1). Of 445 information-only alerts, 28.1% (125/445) were followed by immediate viewing of the patient’s EHR, as were 54.8% (17/31) of the medication-recommendation alerts and 47.1% (66/140) of the test-recommendation alerts.

In multivariate analyses predicting immediate viewing of the patient’s electronic information, the only factor that reached statistical significance was the type of alert. As compared to information-only alerts (reference group), the odds ratio (OR) of immediate action for medication-recommendation alerts was 4.03 (95% CI 1.67-9.72); the OR for test-recommendation alerts was 2.14 (95% CI 1.38-3.32). In the 5 minutes postopening, the mean time PCPs spent on the patient of interest—with the alert on display and while navigating relevant patient’s EHR—was 106 seconds (median, 64 seconds). Alerts not immediately followed by viewing of the relevant patient’s EHR were kept on display for a mean time of 26 seconds (median, 15 seconds; Table 2).

**Table 1:** Immediate electronic health record actions taken by primary care providers after opening noninterruptive alerts. We identified the first time the alert was opened by the addressed primary care physician and extracted from the electronic health record access log table data on the first action taken by the provider during the 5-minute period immediately following alert opening.

Characteristics of alerts, physicians, patients, and contextual factors				N		Immediate action^a^ taken in the record of the patient who triggered the alert, n (%)		No immediate action^b^ taken in the record of the patient who the triggered alert, n (%)
Total				616		208 (33.8)		408 (66.2)
**Alert type**								
	Information only		445		125 (60.1)		320 (78.4)	
	Medication recommendation		31		17 (8.2)		14 (3.4)	
	Test recommendation		140		66 (31.7)		74 (18.1)	
**Time to alert opening**								
	Opened ≤1 hour		119		32 (15.4)		87 (21.3)	
	Opened ≤24 hours		252		88 (42.3)		164 (40.2)	
	Opened >24 hours		245		88 (42.3)		157 (38.5)	
**Characteristics of the** **primary care physician**								
	**Gender**							
		Female	204		72 (34.6)		132 (32.4)	
		Male	412		136 (65.4)		276 (67.6)	
	**Number of patient encounters in the study year (quartiles)^c^**							
		>0 and ≤2326	42		19 (9.1)		23 (5.6)	
		>2326 and ≤2783	115		33 (15.9)		82 (20.1)	
		>2783 and ≤3173	203		70 (33.7)		133 (32.6)	
		>3173	256		86 (41.3)		170 (41.7)	
**Patient characteristic - Charlson comorbidity score**								
		0	58		18 (8.7)		40 (9.8)	
		1	67		20 (9.6)		47 (11.5)	
		≥2	491		170 (81.7)		321 (78.7)	
**Contextual factors**								
	**Opening within office hours**							
		No: Opened outside office hours	312		110 (52.9)		202 (49.5)	
		Yes: Opened 8 AM to 5 PM Mon-Fri	304		98 (47.1)		206 (50.5)	
	**Total number of notifications (opened + unopened) in InBasket at time of alert delivery**							
		≤42	159		42 (20.2)		117 (28.7)	
		>42 and ≤69	152		50 (24)		102 (25)	
		>69 and ≤157	153		62 (29.8)		91 (22.3)	
		>157	152		54 (26)		98 (24)	
	**Number of unopened notifications in InBasket at the time of alert delivery**							
		≤0	189		60 (28.8)		129 (31.6)	
		>0 and ≤4	135		43 (20.7)		92 (22.5)	
		>4 and ≤9	145		50 (24)		95 (23.3)	
		>9	147		55 (26.4)		92 (22.5)	
	**Notification count in the week prior to alert delivery**							
		≤344	146		54 (26)		92 (22.5)	
		>344 and ≤453	159		48 (23.1)		111 (27.2)	
		>453 and ≤546	157		42 (20.2)		115 (28.2)	
		>546	154		64 (30.8)		90 (22.1)	

^a^Actions that we considered as viewing relevant patient information included opening a section of the electronic medical record (medications, laboratory, orders, results, encounters, demographic information, other clinical information, nonclinical information, and information entry) or choosing one of several options on the alert that served as direct links to summaries of components of that patient’s record.

^b^Actions that we considered as not viewing relevant patient information included opening a notification related to a different patient, opening a section of a different patient’s medical record, or doing nothing further in the electronic health record for 5 minutes.

^c^We categorized the total number of patient encounters for each physician during the year prior to alert delivery in quartiles across the 75 primary care physicians to whom alerts were sent to approximate full- vs part-time status.

**Table 2:** Time spent by the primary care physician in electronic charts of patients triggering an alert during the 5 minutes postalert opening.

Time spent in the electronic chart, by category		Time (seconds)	
		Mean	Median
**Time spent in the chart of the relevant patient (n=616 alerts)**		55	22
	Time spent in the chart when the primary care physician took immediate action in the record of the patient who triggered the alert^a^ (n=208)	106	64
	Time spent in the chart when the primary care physician took no immediate action in the record of the patient who triggered the alert^b^ (n=408)	26	15
**Time spent in the chart of the relevant patient, by alert type**			
	Information only (n=445)	42	17
	Medication recommendation (n=31)	81	51
	Test recommendation (n=140)	90	42

^a^Actions that we considered as viewing relevant patient information included opening a section of the electronic medical record (medications, laboratory, orders, results, encounters, demographic information, other clinical information, nonclinical information, and information entry) or choosing one of several options on the alert that served as direct links to summaries of components of that patient’s record.

^b^Actions that we considered as not viewing relevant patient information included opening a notification related to a different patient, opening a section of a different patient’s medical record, or doing nothing further in the EHR for 5 minutes. Thus, this number represents an estimate of time spent viewing an alert.

### Subsequent Relevant Actions

In the overall group of 799 alerts opened, 538 (67.3%) were followed by PCP-related actions during the day of opening or the next day (Table 3). Among information-only alerts, 64.4% (382/593) were followed by a general action in the EHR; 16.0% (95/593), by a medication-specific action; and 16.9% (100/593), by a laboratory-specific action. Among medication-recommendation alerts, 78.4% (29/37) were followed by a general action; 48.6% (18/37), by a medication-specific action; and 54.1% (20/37), by a laboratory-specific action. Among test-recommendation alerts, 75.1% (127/169) were followed by a general action, 24.3% (41/169) were followed by a medication-specific action, and 35.5% (60/169) were followed by a laboratory-specific action.

On multivariate analysis (Table 3), alerts containing specific instructions for the PCP (medication recommendations or test recommendations) were significantly more likely to be associated with subsequent action by the PCP compared to information-only alerts. Compared to information-only alerts, the ORs of medication-specific action were significantly greater for medication recommendations (OR: 5.59; 95% CI 2.42-12.94) and test recommendations (OR: 1.71; 95% CI 1.09-2.68). Likewise, the ORs of laboratory-specific action were significantly greater for medication recommendations (OR: 7.37; 95% CI 3.64-14.97) and test recommendations (OR: 2.75; 95% CI 1.73-4.38).

There was no significant association between the timing of alert opening and responsive action. As expected, subsequent relevant action by the PCP was more likely for alerts opened by the addressed PCP as compared to alerts opened by staff or another provider, although only the association with medication-specific action reached statistical significance (general action, OR: 1.41; 95% CI 0.81-2.45; medication-specific action, OR: 2.13; 95% CI 1.35-3.37; laboratory-specific action, OR: 1.65; 95% CI 0.99-2.73).

**Table 3:** Multivariate analysis results for factors associated with primary care providers’ subsequent action following opening of noninterruptive alerts. Alerts were opened by the addressed primary care provider, staff, or provider other than the addressed primary care provider. The primary care provider’s actions in the electronic health record related to the relevant patient were tracked over the day of alert opening and the following day.

Characteristics of alerts, physicians, patients, and contextual factors				Total alerts		General action^a^ in electronic health record taken by PCP^b^				Medication-specific action^c^ taken by PCP				Laboratory-specific action^d^ taken by PCP		
				N (%)		Action is taken, N (%)		AOR^e^ (95% CI)		Action is taken, N (%)		AOR (95% CI)		Action is taken, N (%)		AOR (95% CI)
Total				799		538 (67.3)		N/A^f^		154 (19.3)		N/A		180 (22.5)		N/A
**Alert type**																
	Information only		593 (74.2)		382 (71)		Reference		95 (61.7)		Reference		100 (55.6)		Reference	
^ ^	Medication recommendation^g^		37 (4.6)		29 (5.4)		2.0 (0.9- 4.8)		18 (11.7)		5.6 (2.4- 12.9)		20 (11.1)		7.4 (3.6- 15.0)	
^ ^	Test recommendation^h^		169 (21.2)		127 (23.6)		1.7 (1.1- 2.5)		41 (26.6)		1.7 (1.1- 2.7)		60 (33.3)		2.8 (1.7- 4.4)	
**Time to alert opening**																
	Opened ≤24 h after delivery		472 (59.1)		308 (57.2)		Reference		88 (57.1)		Reference		101 (56.1)		Reference	
	Opened >24 h and ≤48 h after delivery		137 (17.1)		97 (18)		1.2 (0.6- 2.4)		27 (17.5)		1.2 (0.7- 1.9)		28 (15.6)		1.1 (0.6- 2.0)	
	Opened >48 h after delivery		190 (23.8)		133 (24.7)		1.2 (0.7- 2.2)		39 (25.3)		1.1 (0.6- 1.8)		51 (28.3)		1.4 (0.8- 2.4)	
**Opened by PCP/other staff**																
	Opened by staff/provider other than PCP		172 (21.5)		100 (18.6)		Reference		17 (11)		Reference		23 (12.8)		Reference	
	Opened by PCP		627 (78.5)		438 (81.4)		1.4 (0.8-2.5)		137 (89)		2.1 (1.4-3.4)		157 (87.2)		1.7 (1.0-2.7)	
**PCP characteristics**																
	**Age**															
		<50 years	296 (37.0)		203 (37.7)		Reference		63 (40.9)		Reference		80 (44.4)		Reference	
		≥50 years	503 (63.0)		335 (62.3)		1.0 (0.7- 1.4)		91 (59.1)		0.6 (0.4- 1.1)		100 (55.6)		0.6 (0.4- 0.9)	
	**Gender**															
		Female	264 (33.0)		182 (33.8)		Reference		54 (35.1)		Reference		71 (39.4)		Reference	
		Male	535 (67.0)		356 (66.2)		0.6 (0.4- 1.0)		100 (64.9)		0.7 (0.4- 1.3)		109 (60.6)		0.5 (0.3- 0.7)	
	**Number of patient encounters in study year (quartiles)^i^**															
		>0 and ≤2326	80 (10.0)		58 (10.8)		Reference		8 (5.2)		Reference		14 (7.8)		Reference	
		>2326 and ≤2783	163 (20.4)		94 (17.5)		0.6 (0.3- 1.4)		18 (11.7)		0.5 (0.4- 2.8)		30 (16.7)		1.0 (0.4- 2.2)	
		>2783 and ≤3173	247 (30.9)		168 (31.2)		1.0 (0.5- 2.1)		54 (35.1)		1.5 (1.2- 8.0)		59 (32.8)		1.9 (0.9- 4.0)	
		>3173	309 (38.7)		218 (40.5)		1.5 (0.7- 3.3)		74 (48.1)		2.5 (1.5- 13.3)		77 (42.8)		2.7 (1.3- 5.8)	
	**Specialty**															
		Internal medicine	118 (14.8)		445 (82.7)		Reference		129 (83.8)		Reference		155 (86.1)		Reference	
		Family medicine	661 (82.7)		79 (14.7)		0.7 (0.4- 1.2)		22 (14.3)		0.9 (0.4- 1.8)		20 (11.1)		0.6 (0.4- 0.9)	
		Non-MD^j^ PCP	11 (1.4)		6 (1.1)		0.3 (0.1- 1.2)		2 (1.3)		0.6 (0.2- 1.9)		3 (1.7)		0.6 (0.3- 1.4)	
		Subspecialty	9 (1.1)		8 (1.5)		3.0 (1.0- 9.6)		1 (0.6)		1.4 (0.4- 5.3)		2 (1.1)		2.1 (0.7- 6.0)	
**Patient characteristics**																
	**Age**															

		65-74 years	255 (31.9)		171 (31.8)		Reference		55 (35.7)		Reference		56 (31.1)		Reference	
		75-84 years	349 (43.7)		241 (44.8)		1.1 (0.7- 1.6)		57 (37)		0.8 (0.5- 1.1)		77 (42.8)		1.0 (0.6- 1.4)	
		≥85 years	195 (24.4)		126 (23.4)		0.9 (0.5- 1.4)		42 (27.3)		1.0 (0.6- 1.5)		47 (26.1)		1.0 (0.6- 1.6)	
	**Gender**															
		Female	418 (52.3)		284 (52.8)		Reference		81 (52.6)		Reference		93 (51.7)		Reference	
		Male	381 (47.7)		254 (47.2)		0.9 (0.7- 1.3)		73 (47.4)		1.1 (0.8- 1.6)		87 (48.3)		1.3 (0.9- 1.8)	
	**Number of office visits in the previous 12 months**															

		≤6 visits	205 (25.7)		125 (23.2)		Reference		41 (26.6)		Reference		39 (21.7)		Reference	
		>6 and ≤11 visits	219 (27.4)		142 (26.4)		1.3 (0.9- 2.0)		36 (23.4)		0.9 (0.5- 1.6)		44 (24.4)		1.0 (0.6- 1.7)	
		>11 and ≤18 visits	194 (24.3)		144 (26.8)		2.1 (1.3- 3.6)		42 (27.3)		1.4 (0.8- 2.3)		48 (26.7)		1.4 (0.6- 1.7)	
		>18 visits	181 (22.7)		127 (23.6)		1.7 (1.0- 2.7)		35 (22.7)		1.2 (0.6- 2.1)		49 (27.2)		1.5 (0.9- 2.5)	
	**Charlson comorbidity score**															
		0	78 (9.8)		51 (9.5)		Reference		17 (11)		Reference		11 (6.1)		Reference	
		1	92 (11.5)		56 (10.4)		0.7 (0.3- 1.4)		16 (10.4)		0.7 (0.3- 1.7)		17 (9.4)		1.3 (0.5- 3.1)	
		2	118 (14.8)		80 (14.9)		0.9 (0.4- 1.8)		21 (13.6)		0.7 (0.3- 1.6)		25 (13.9)		1.6 (0.6- 3.9)	
		≥3	511 (64.0)		351 (65.2)		0.8 (0.4- 1.4)		100 (64.9)		0.6 (0.3- 1.3)		127 (70.6)		1.3 (0.6- 2.8)	
	**Length of stay**															
		≤2 days	360 (45.1)		229 (42.6)		Reference		65 (42.2)		Reference		74 (41.1)		Reference	
		3 days	281 (35.2)		199 (37)		1.4 (1.0- 2.1)		57 (37)		1.1 (0.8- 1.6)		67 (37.2)		1.2 (0.9- 1.7)	
		≥4 days	158 (19.8)		110 (20.4)		1.5 (0.9- 2.5)		32 (20.8)		1.3 (0.8- 2.1)		39 (21.7)		1.5 (0.9- 2.4)	
**Contextual factors**																
	**Total number of notifications in InBasket at the time of alert delivery**															
		≤42	207 (25.9)		146 (27.1)		Reference		43 (27.9)		Reference		51 (28.3)		Reference	
		>42 and ≤69	194 (24.3)		134 (24.9)		0.9 (0.5- 1.5)		32 (20.8)		0.9 (0.5- 1.5)		34 (18.9)		0.7 (0.5- 1.1)	
		>69 and ≤157	199 (24.9)		135 (25.1)		0.7 (0.4- 1.2)		37 (24)		1.0 (0.6- 1.8)		51 (28.3)		1.4 (0.9- 2.0)	
		>157	199 (24.9)		123 (22.9)		0.5 (0.3-1.0)		42 (27.3)		1.0 (0.7- 2.4)		44 (24.4)		0.9 (0.6- 1.5)	
	**Number of unopened notifications in InBasket at the time of alert delivery**															
		≤0	251 (31.4)		165 (30.7)		Reference		50 (32.5)		Reference		57 (31.7)		Reference	
		>0 and ≤4	183 (22.9)		122 (22.7)		1.0 (0.7- 1.5)		34 (22.1)		0.8 (0.5-1.3)		42 (23.3)		0.9 (0.6- 1.5)	
		>4 and ≤9	185 (23.2)		127 (23.6)		1.2 (0.7- 2.0)		32 (20.8)		0.7 (0.5- 1.0)		45 (25)		0.8 (0.5- 1.4)	
		>9	180 (22.5)		124 (23)		1.5 (0.9- 2.5)		38 (24.7)		1.2 (0.8- 1.8)		36 (20)		0.8 (0.5- 1.2)	
	**Notification count in the week prior to alert delivery**															
		≤344	200 (25.0)		146 (27.1)		Reference		39 (25.3)		Reference		55 (30.6)		Reference	
		>344 and ≤453	201 (25.2)		127 (23.6)		0.7 (0.4- 1.0)		43 (27.9)		0.9 (0.6- 1.4)		49 (27.2)		0.6 (0.4- 1.1)	
		>453 and ≤546	199 (24.9)		135 (25.1)		0.7 (0.5- 1.1)		35 (22.7)		0.6 (0.4- 0.9)		35 (19.4)		0.4 (0.3- 0.8)	
		>546	199 (24.9)		130 (24.2)		0.7 (0.4- 1.1)		37 (24)		0.6 (0.4- 1.0)		41 (22.8)		0.6 (0.4- 1.0)	
	**Day of the week alert sent**															
		All other days	633 (79.2)		418 (77.7)		Reference		119 (77.3)		Reference		143 (79.4)		Reference	
		Saturday	166 (20.8)		120 (22.3)		1.3 (0.7- 2.2)		35 (22.7)		1.2 (0.7- 1.9)		37 (20.6)		0.9 (0.6- 1.6)	

^a^General Action in electronic health record includes opening a section of the electronic medical record (medications, laboratory, orders, results, encounters, demographic information, other clinical information, nonclinical information, and information entry) or choosing one of several options on the alert that served as direct links to summaries of components of that patient’s record.

^b^Medication-specific action includes medication list viewing and ordering.

^c^Laboratory-specific action includes laboratory viewing and ordering.

^d^PCP: primary care physician.

^e^AOR: adjusted odds ratio.

^f^N/A: not applicable.

^g^Medication recommendations were automated electronic health record InBasket alerts that contained warnings about interactions or recommendations for dose changes.

^h^Test recommendations were automated electronic health record InBasket alerts identifying the need for laboratory monitoring for high-risk medications.

^i^We categorized the total number of patient encounters for each physician during the year prior to alert delivery in quartiles across the 75 primary care physicians to whom alerts were sent to approximate full- vs part-time status.

^j^MD: doctor of medicine.

Contextual factors showed no consistent patterns. There was some suggestion of a stronger likelihood for responsive action if the relevant patient had more than 11 office visits in the past year. Responsive actions were also more likely taken by physicians with more patient encounters and less likely by physicians with total number of InBasket notifications at the time of alert delivery in the top quartile (>157 alerts).

### Electronic Health Record Access and Audit Logs

#### Challenges

We were able to identify all the data that were required to track provider behavior in the EHR access and audit logs; however, a substantial effort was required to identify and interpret these data. We encountered two main challenges in data retrieval and interpretation. As detailed below, these challenges pertained to capturing a historical snapshot (eg, number of InBasket messages at a given time in the past) and interpreting data elements from access and audit logs in the absence of clear data documentation.

#### Capturing a Historical Snapshot of Electronic Health Record Status

Our access logs do not keep successive records of evolving EHR elements (examples include InBasket message lists or medication lists, both of which have elements added and deleted over time). Thus, in order to capture the status of a provider’s InBasket retrospectively, we had to retrieve data generated many months prior and couple this with longitudinal follow-up data to determine the status at the time of interest. Specifically, to recreate a list of InBasket messages present at the time of alert delivery, we obtained an estimate of the date on which the earliest arriving messages were generated (we went back 1 year) and then captured all the messages generated from that point forward. We honed this large list using status data reflecting whether the message was opened and whether it had been “completed” (eg, deleted) prior to the date of alert delivery. Messages that were marked as completed (and those postponed to return at a future date) were removed; the remaining were concluded to be messages present in the InBasket at the time of alert delivery.

#### Interpretation of Data Elements Required an Iterative Approach With Physician-Data Manager Collaboration

We encountered challenges in deciphering the variable names for data elements drawn from the audit logs. These data had not previously been used for research purposes, and therefore, we lacked clear documentation for the interpretation of many variables. Accurately interpreting data elements required iterative work by the physician/data manager team. Using a simulated patient record in the EHR, our physician collaborator performed actions through the physician user interface and then worked with the data manager to compare results captured simultaneously through the audit log. Both a physician’s perspective (providing insight into routine actions taken by physicians within an EHR) and the data manager’s informatics knowledge (and access to data tables) were essential for this effort.

## Discussion

Our descriptive study assessed the use of EHR log data to track provider behavior following opening of noninterruptive medication alerts. More than three-quarters of alerts (78.5%; 627/799) were opened by the PCP to whom they were directed, allowing us to assess immediate PCP action; of these, 208 alerts were followed by immediate action. Expanding our analysis to include alerts opened by staff or covering physicians, we found that an additional 330/799 alerts showed evidence of action by the end of the following day. The remaining 261 alerts showed no evidence of PCP action. Compared to information-only alerts, alerts containing specific instructions (including medication and test recommendations) were significantly more likely to be followed by EHR action; these alerts were also more likely to prompt medication-specific action such as medication ordering, changing, and discontinuing or medication list viewing.

Not all alerts were followed by provider action, indicating that other factors may have played a role in providers’ decision to act on information or recommendations provided in the alerts.

Data from access and audit logs provide a rare “behind-the-scenes” glimpse into how health care team members spend their time and where they direct their attention. These log data have been used successfully to understand questions of the health care team’s communication [26], trainee skill [27], and clinical workflow [6,7]. As we demonstrate in this paper, EHR logs can also provide valuable implementation data that can be used to improve future CDS interventions. Log data can help a research team understand whether a CDS message was opened by the addressed recipient, how long it was viewed for, and what kind of actions followed the viewing.

There are numerous ways a research team can benefit from this knowledge. Our finding that few physician, patient, and contextual aspects impacted the opening of and action on alerts is reassuring. It does not appear that subgroups of PCPs or patients would require varying approaches for alert-based communication. Tracking rates of opening for noninterruptive messages can identify a need for improved safety measures (eg, escalation of unopened time-sensitive messages). Understanding who opens these messages lends insight into a care team’s triage processes, which might alter the design of the intervention (eg, physician-directed CDS sent to a team where all messages are opened initially by nurses might prompt the team to modify message content or change the method of communication) [4]. Realizing that providers spend an extremely short period of time with alerts open might prompt those designing these alerts to adapt messages accordingly (eg, include fewer words and optimize visibility of key text). For the intervention discussed in this paper, we preceded implementation by having two of the group practice primary care physicians review all messages generated by the system for four months. Of the tested alert types, the reviewers selected those included in the final intervention with the goal that all alerts would be necessary and actionable. For the medication information-only alerts, which focused specifically on new or high-risk medications, the reviewers were certain that recipients would review the full medication list for these patients. Alerts that were categorized as information-only may have been perceived by PCPs as not requiring further action, since they did not include specific recommendations. The brief viewing time and low level of activity after viewing information-only alerts suggests that this type of alert should be reconsidered before including them in future interventions. Tracking patterns of EHR behavior after alert opening might suggest useful shortcuts to make the alert more user-friendly (eg, if users routinely navigate to the medication list after opening the alert, providing a link directly to this destination might enhance the user experience). In sum, to understand why an EHR–based CDS intervention is (or is not) effective, researchers should be asking whether that intervention was delivered as planned and what subsequent steps were taken by recipients after intervention delivery.

In this descriptive study, we focused on use of access and audit logs for understanding the impact of noninterruptive alerts. This approach is particularly necessary for studying noninterruptive alerts, which are usually opened outside of a patient encounter and thus present challenges for those seeking to pinpoint the timing of downstream actions. Access and audit log data can also be applied to a variety of questions pertaining to transmission of EHR–based information. Examples include tracking of information viewing (eg, understanding when a care team member opens and reads a clinic note, laboratory or test report, or other updated EHR information) as well as tracking the timing of documentation and ordering.

Access and audit logs could also be helpful in trying to reduce the InBasket burden for physicians. Log data can reveal which types of alerts and notifications physicians act upon; those that are commonly left unopened or are not generally followed by any action could be candidates for elimination or modification. These data can also inform enhancements to the structure of the InBasket. Unopened or unacted upon messages can be moved to and organized within separate folders to which the providers can return at a later time.

In addition to the two challenges identified here (difficulties in capturing a historical snapshot from the EHR and difficulty in interpretation of data elements in the absence of clear documentation), there are additional issues to consider when using access and audit log data. Site-to-site variability in the use of certain variables may introduce differences in interpretation of audit logs across sites. Data managers may not have privileges granted to retrieve access or audit log data. The large volume of data generated through access and audit logs may necessitate policies for limitations in what a site stores (and for how long). Understanding the local situation at an early stage of research planning may facilitate use (for instance, planning to download log data before they are deleted at a site that stores them for only a limited period). Planning ahead and setting up log data to provide real-time information as an intervention is implemented can make the process much simpler.

There are limitations to this study. We may not have captured all PCP actions after alert opening relevant to the postdischarge patient, such as actions taken outside of the EHR. The age of the data (from a trial conducted in 2011) may not accurately reflect current provider workflow in upgraded versions of the EHR with respect to opening of alerts and responsive actions. Direct causality (EHR action due to alert opening) is not proven simply by demonstrating close temporal proximity. Our study is descriptive and could be supported by future qualitative work examining the physicians’ own perspectives on their EHR actions following alert opening.

An EHR–based alert system intervention alone does not improve clinical patient outcomes in high-risk populations. Nonetheless, the availability of information in the EHR can benefit similar studies trying to understand the link between provider behavior in the EHR and patient care and outcomes. Although analysis of EHR logs presents many challenges, data from these logs can provide researchers with insight into designing impactful EHR–based CDS and alert interventions.

## Abbreviations

AOR: adjusted odds ratio
CDS: clinical decision support
EHR: electronic health record
N/A: not applicable
OR: odds ratio
PCP: primary care physician
